# The specificity of the interaction between *α*B-crystallin and desmin filaments and its impact on filament aggregation and cell viability

**DOI:** 10.1098/rstb.2012.0375

**Published:** 2013-05-05

**Authors:** Jayne L. Elliott, Ming Der Perng, Alan R. Prescott, Karin A. Jansen, Gijsje H. Koenderink, Roy A. Quinlan

**Affiliations:** 1 School of Biological and Biomedical Sciences, The University of Durham, South Road, Durham DH1 3LE, UK; 2 Biophysical Sciences Institute, The University of Durham, South Road, Durham DH1 3LE, UK; 3 Institute of Molecular Medicine, College of Life Sciences, National Tsing Hua University, Hsinchu 300, Taiwan, Republic of China; 4 CHIPs and Division of Cell Signalling and Immunology, College of Life Sciences, University of Dundee, WTB/MSI complex, Dundee DD1 5EH, UK; 5 FOM Institute AMOLF, Science Park 104, Amsterdam XG 1098, The Netherlands

**Keywords:** αB-crystallin/CRYAB/HSPB5, desmin-related myopathy, desmin intermediate filament, crystallinopathy, cardiomyopathy

## Abstract

CRYAB (*α*B-crystallin) is expressed in many tissues and yet the R120G mutation in CRYAB causes tissue-specific pathologies, namely cardiomyopathy and cataract. Here, we present evidence to demonstrate that there is a specific functional interaction of CRYAB with desmin intermediate filaments that predisposes myocytes to disease caused by the R120G mutation. We use a variety of biochemical and biophysical techniques to show that plant, animal and ascidian small heat-shock proteins (sHSPs) can interact with intermediate filaments. Nevertheless, the mutation R120G in CRYAB does specifically change that interaction when compared with equivalent substitutions in HSP27 (R140G) and into the *Caenorhabditis elegans* HSP16.2 (R95G). By transient transfection, we show that R120G CRYAB specifically promotes intermediate filament aggregation in MCF7 cells. The transient transfection of R120G CRYAB alone has no significant effect upon cell viability, although bundling of the endogenous intermediate filament network occurs and the mitochondria are concentrated into the perinuclear region. The combination of R120G CRYAB co-transfected with wild-type desmin, however, causes a significant reduction in cell viability. Therefore, we suggest that while there is an innate ability of sHSPs to interact with and to bind to intermediate filaments, it is the specific combination of desmin and CRYAB that compromises cell viability and this is potentially the key to the muscle pathology caused by the R120G CRYAB.

## 1. Introduction

The discovery that the R120G mutation in αB-crystallin (CRYAB, HSPB5 [1]) phenocopies desmin mutations [2,3] in human desmin-related myopathies (DRMs) provided the first genetic evidence in support of the proposed functional interaction between CRYAB and intermediate filaments [4]. Since that discovery, there have been many studies on the mechanisms that cause DRM. These have included the identification of the amyloid-forming potential of CRYAB [5,6], the involvement of the proteosomal [7] and macroautophagy pathways [8], as well as the propagation of apoptotic signals via desmin fragmentation [9] and the involvement of mitochondria [10]. Desmin is intimately involved in mitochondrial positioning and homeostasis [11–15] and mitochondrial changes are a prominent associated phenotype in both DRM patients [16] and mouse models of cardiomyopathy [17]. The caspase 6-mediated fragmentation of desmin produces an *N*-terminal fragment that promotes filament aggregation [9]. Blocking this has been shown to attenuate another model of cardiomyopathy based on tumour necrosis factor-mediated apoptosis [18]. Other mouse models of cardiomyopathy that have not genetically targeted desmin or CRYAB expression [19,20] see changes in desmin distribution and its inclusion into aggregates and an association with CRYAB. The emerging consensus is that the redistribution of desmin into aggregates [21,22] is a key initiator in the pathology of DRMs.

Protein aggregates containing both desmin and CRYAB were a feature of the description of the R120G CRYAB family [1]. This was faithfully replicated in a knock-in mouse model of the R120G CRYAB disease-causing mutation, with desmin aggregation in muscles and the additional observation of increased vimentin aggregation in the lenses of these animals [23]. Cataracts were also noted in those family members expressing R120G CRYAB [1]. These data could suggest that the CRYAB interaction may not be specific to desmin, as vimentin also associates with CRYAB [4]. Indeed, the co-association of CRYAB with intermediate filament aggregates is a common histopathological feature in the human diseases caused by mutant cytoplasmic intermediate filament proteins that form aggregates [24]. Therefore, it is important to examine the specificity of the functional interaction between desmin filaments and CRYAB if we are to understand fully the muscle pathology caused by mutant CRYAB and desmin.

Previously, we have shown that small heat-shock proteins (sHSPs) are important modulators of intermediate filament assemblies [4]. They can prevent filament–filament interactions from occuring on the basis of an *in vitro*-based viscosity assay and transient transfection studies [25]. The R120G mutation in CRYAB was found to abrogate this activity both for glial fibrillary acidic protein (GFAP) [25] and desmin filaments [26], promoting instead filament–filament interactions and their aggregation. Mutations in other sHSPs also cause human diseases from cataract to distal neuropathies, which include CRYAA (HSPB4), HSP27 (HSPB1), HSP27L (HSPB3; [27]) and HSP22 (HSPB8) (summarized in [28]). Intermediate filament aggregates feature in the histopathologies of such diseases, demonstrating that the interaction between sHSPs and intermediate filaments is a widespread and functionally important interaction. The question to emerge from these studies is why only certain cell types and tissues are affected by mutations in an sHSP, whereas their tissue expression profile is not usually restricted (except perhaps CRYAA [29]). CRYAB [30], HSP27 and HSP22 are all expressed in muscle [31–33], but mutations in HSP27 and HSP22 are not associated with muscle pathology.

There have been many suggestions to explain the tissue-specific pathologies associated with sHSP mutations [34–40], but we propose that the specific intermediate filament expression pattern must be considered as a key factor in any sHSP-based pathology as we consider the intermediate filament–sHSP complex to be a functional unit [41,42]. Intermediate filament expression profiles follow tissue-specific patterns according to embryological origins [43]. It has already been shown that R120G CRYAB induces the aggregation of desmin filaments [26], but it can also potentially cause the aggregation of GFAP filaments [25]. The reported pathologies for CRYAB mutations are, however, myopathies and cataract and not neuropathies. Desmin is a type III intermediate filament protein expressed in muscle, which suggests that the interaction of desmin with CRYAB is key to understanding DRM.

To test this hypothesis, we considered the consequences of introducing CRYAB R120G equivalent mutations into other sHSPs, such as HSP27 and HSP16.2, to see if equivalent mutations would also change their interaction with desmin. The R120G mutation in the α-crystallin domain of CRYAB is predicted to have similar structural consequences for HSP27 [44], and therefore for both HSP27 and CRYAB the equivalent mutation should have similar effects. We find that only the R120G CRYAB mutation induces increased binding to desmin as assessed by *in vitro* sedimentation assay. We assessed the interaction of desmin and CRYAB using a range of *in vitro* techniques (falling ball assay, Ostwald viscometry, surface plasmon resonance (SPR) and optical trap measurement of filament network elasticity) to evidence the interaction of CRYAB with desmin. We show that the binding of CRYAB to desmin is pH- and cation-dependent. Using transient transfection, we show that only the desmin-CRYAB R120G combination-induced desmin aggregates coincided with reduced cell viability in MCF7 cells. We suggest that it is the partnership of the sHSP with the resident intermediate filaments that determines how cells respond to the presence of mutant CRYAB.

## 2. Material and methods

### (a) Expression constructs for recombinant sHSPs

Wild-type (WT) or R120G CRYAB expression vectors based on the pET23b plasmid were constructed as described previously [25]. HSP27 and R140G HSP27 were constructed as described [45]. The *Caenorhabditis elegans* HSP16.2 cDNA was cloned into the pRSET expression vector (Invitrogen) as described previously [46] using the QuickChange site-directed mutagenesis kit (Stratagene) to introduce the R95G mutation into WT HSP16.2. For live cell imaging experiments, CRYAB or desmin were subcloned into the modified pcDNA3.1 (+) vector with DsRed2-Mito (Clontech) preceded by an internal ribosomal entry site (IRES). These two vector components were PCR amplified from the vectors DsRed2-Mito (Clontech) and pWPI (http://tronolab.epfl.ch) and sequenced in pGEM-T Easy (Promega, UK) before assembling with the relevant CRYAB or desmin fragments from the pET23. These IRES-containing bicistronic vectors allow simultaneous expression of both mitochondrially targeted red fluorescent protein to indicate transfected cells and either CRYAB or desmin constructs.

### (b) Expression and purification of recombinant wild-type and mutant sHSPs

Both WT and mutant sHSPs were expressed in and purified from BL21(DE3) pLysS *Escherichia coli* as described. WT and R120G CRYAB were purified as described using two diethylaminoethanol (DEAE) column steps at 4°C [25]. Recombinant human WT and R140G HSP27 were purified using similar procedures. For further studies, purified sHSPs were refolded by dialysis against 20 mM Tris–HCl, pH 7.4, 100 mM NaCl at 4°C for 16 h.

Both the WT and R95G HSP16.2 formed inclusion bodies, which were purified [47] and then solubilized in TEN buffer containing 8 M urea. Purification required anion exchange chromatography using DEAE-cellulose (DE52; Whatman, UK) in the presence of 6 M urea. Peak fractions were pooled and then dialysed against buffer containing 20 mM Tris–HCl, pH 7.4, 100 mM NaCl. The native complex was further purified by size exclusion chromatography (SEC) on a Fractogel EMD BioSEC Superformance column (60 × 1.6 cm; Merck, UK) in the same buffer. Purified proteins were concentrated to 1 mg ml^−1^ using Ultrafree-15 (Millipore, UK) concentrators with a 10 kDa molecular weight cut-off.

### (c) Preparation of desmin, glial fibrillary acidic protein and keratins

Purified desmin was obtained by extraction of the crude intermediate filament preparation from chicken gizzards with 8 M urea and the subsequent chromatography on DEAE-cellulose and hydroxyapitite columns in the presence of 6 M urea as described previously [48,49]. Recombinant human desmin, GFAP, keratins 7 and 18 were purified as described [4,26,50,51]. Protein concentrations were determined by the bicinchonic acid assay (BCA reagent, Pierce) using bovine serum albumin as standard.

### (d) Size exclusion chromatography of sHSPs

Molecular size of the recombinant sHSP complexes were measured by gel filtration chromatography on a Superformance column (60 × 1.6 cm) packed with Fractogel EMD BioSEC (Merck, UK). The column was calibrated using thyroglobulin (669 kDa), apoferritin (440 kDa), alpha-amylase (200 kDa), bovine serum albumin (67 kDa) and carbonic anhydrase (29 kDa). The column void volume was determined using dextran blue (2000 kDa). Proteins were eluted in buffer containing 20 mM Tris–HCl, pH 7.4 and 100 mM NaCl at room temperature and the elution volume of each sample was used to estimate the molecular weight.

### (e) Intermediate filament assembly, binding and viscosity assays involving sHSPs

Low-speed and high-speed sedimentation assays were used to assess the ability of sHSPs to associate with intermediate filaments and prevent filament–filament associations that lead to aggregation [52]. Intermediate filament proteins were mixed with sHSPs in urea buffer (8 M urea, 20 mM Tris–HCl, pH 8.0, 5 mM EDTA, 2 mM EGTA, 1 mM DTT) and then dialysed to lower the urea concentration stepwise into low ionic strength buffer (10 mM Tris–HCl pH 7.0, 1 mM DTT) at 4°C. Sometimes CRYAB was added at this stage prior to initiating filament assembly by dialysis into filament assembly buffer (10 mM Tris–HCl pH 7.0, 1 mM DTT 50 mM NaCl) at room temperature for 12 h. Assembly of desmin and GFAP filaments was also initiated by the addition of a 20-fold concentrated binding buffer to low ionic strength buffer, giving a final concentration of 100 mM imidazole-HCl, pH 6.8, 1 mM DTT. Protein samples were incubated for 2 h at the indicated temperatures. Experiments to investigate pH and temperature effects on CRYAB associations were carried out as follows with WT GFAP, vimentin and desmin assembled at 0.2 mg ml^−1^ and mixed with WT CRYAB at a 1 : 1 molar ratio. Filament assembly was completed by dialysis into 20 mM *N*-2-hydroxyethylpiperazine-*N*-2-ethanesulfonic acid (HEPES), 100 mM NaCl, 1 mM MgCl_2_, 1 mM DTT at pH 6.3, 6.8 or 7.3; at 23°C, 39°C or 44°C, respectively. In some instances, CRYAB was also added to assembled filaments.

The influence of sHSPs on filament–filament interactions of assembled desmin was assessed also by measuring the viscoelastic properties. First, we used a falling ball assay as described previously [51]. The ability of the sample to support a ball bearing was then scored in a binary fashion. Carbonic anhydrase (Sigma, UK) was used as a control. We performed viscosity measurements using an Ostwald-type viscometer (Cannon, USA) at a protein concentration of 0.5 mg ml^−1^ at 37°C. GFAP was assembled in the absence or the presence of CRYAB by addition of a 20-fold binding buffer as described above. Flow times were measured at different time points: 1 min after assembly start and then every 5 min over a period of 1 h. Specific viscosity (*V*_sp_) was calculated by the equation *V*_sp_ = (*T*_s_−*T*_b_)/*T*_b_, where *T*_s_ is the flow time of the sample and *T*_b_ the flow time of the buffer.

### (f) Passive microrheology measurements

Desmin filaments were assembled from purified recombinant protein at 1 mg ml^−1^ with or without a 1 : 10 molar ratio of desmin : CRYAB using dialysis to lower the urea concentration. Assembly was completed by overnight dialysis into 20 mM Tris–HCl (pH 7.3), 50 mM NaCl, 1 mM MgCl_2_ and 1 mM DTT at room temperature. One-particle passive microrheology was done using a 808 nm laser and a 100× oil objective (NA 1.4) on an inverted Nikon phase microscope. PLL-coated polystyrene beads of 1.5 µm were trapped, laser light was collected using an oil condenser, and the intensity fluctuations were recorded using a quadrant photodiode (QPD). With a custom-written program in C++, the apparent elastic modulus G′_app_ and apparent viscous modulus G″_app_ were determined from the fluctuations in bead position using the fluctuation–dissipation theorem and generalized Stokes–Einstein equation [53]. The viscous modulus was calculated from G″_app_ by subtracting the solvent viscosity, and the elastic modulus was calculated from G′_app_ by subtracting the apparent modulus in buffer to compensate for the presence of the optical trap [54].

### (g) Binding of CRYAB and R120G CRYAB measured by surface plasmon resonance

Affinities of WT or R120G CRYAB to immobilized intermediate filament proteins were determined using SPR analysis with a Biacore 3000 apparatus (GE Healthcare, Uppsala, Sweden). Purified desmin, GFAP and vimentin were immobilized on the dextran matrix of a CM5 sensor chip according to the manufacturer's instructions using 10 mM HEPES, pH 7.4, 0.15 M NaCl, 3.4 mM EDTA, 0.005 % (v/v) surfactant P20. Unreacted groups were subsequently blocked by injection of 1 M ethanolamine, pH 8.5. WT and R120G CRYAB for binding to immobilized intermediate filament proteins were first diluted to 20 g ml^–1^, injected at a flow rate of 5 l min^−1^ for 7 min at 37°C and then washed for 7 min. All sensograms were corrected for non-specific interactions to a reference surface and by double referencing [55]. The sensor chip was regenerated between injections by washing with 6 M guanidine hydrochloride in HBS-EP buffer.

### (h) Cell cultures, transient transfection and cell viability assays

The immortalized human lens epithelial cell line H36CE2 was grown as detailed previously [56]. Baby hamster kidney (BHK21) cells, mouse myoblast C2C12 cells and human breast cancer epithelial cell MCF7 were grown in media as recommended by the ECACC (www.ecacc.org.uk). For co-transfection experiments, plasmid DNA (pcDNA3.1; Invitrogen, UK) containing human desmin, CRYAB, HSP27 or HSP16.2 in pcDNA3.1 (Invitrogen, UK) were prepared using MaxiPrep kits (Qiagen, UK). H36CE2 cells were transiently co-transfected by calcium phosphate precipitation using standard procedures [57], while GeneJuice Transfection Reagent (Merck Millipore, UK) was used to transiently transfect the other cell lines. Cells were allowed to recover for 24–48 h prior to processing for immunofluorescence microscopy as described previously [58]. Quantification of the desmin filament phenotypes was performed by visual assessment of various staining patterns in transfected cells. For each DNA construct, cells on three coverslips were counted and approximately 100–150 transfected cells were assessed per coverslip. For cell viability assay, the colorimetric CellTiter 96 AQueous One Solution Cell Proliferation Assay (Promega, UK) was used according to the manufacturer's instructions. Statistical significance was analysed by one-way ANOVA and the level of significance was set at *p* ≤ 0.05. Apoptotic cells were assessed by staining with a monoclonal antibody M30 (1 : 10, Roche Diagnostics, Mannheim, Germany) that specifically recognizes a neo-epitope of keratin 18 fragment generated by caspase cleavage at position Asp^396^ and counterstained with 5 μg ml^−1^ DAPI (Molecular Probe Inc., Eugene, OR, USA).

### (i) Primary antibodies

The primary antibodies used in this study were rabbit polyclonal anti-desmin (1 : 100; Sigma, UK), desmin monoclonal (D3; Developmental Biology Hybridoma Service), mouse monoclonal anti-HSP27 (1 : 100; [59]), mouse monoclonal anti-CRYAB (1 : 1; [60]) or mouse polyclonal anti-HSP16.2 (1 : 50; [46]). Desmin (PDE; Euro-Diagnostica, The Netherlands) and mouse monoclonal anti-desmin (D33; DakoCytomation), keratin (LE41; mouse monoclonal) and GFAP (polyclonal 3270 and monoclonal GA5) were as described [58]. After washing with PBS/BSA/azide, the primary antibodies were detected using FITC (1 : 100; Sigma, UK), Texas-Red (1 : 200; Jackson ImmunoResearch Laboratories, UK) or Alexa 594 (1 : 500; Molecular Probe, UK) conjugated secondary antibodies.

### (j) Preparation of cell lysates and immunoblotting analysis

Cells plated at a density of 1 × 10^6^ cells per 100 mm Petri dish were transfected with expression vectors as indicated. After 48 h, cell lysates were prepared [26] and analysed by immunoblotting followed by enhanced chemiluminescence using a luminescent image analyser (LAS-1000plus; FujiFilm, Japan).

### (k) Live cell imaging and movie preparation

For live cell imaging, cells transfected with pcDNA3.1-IRES-DsRED2-Mito vectors were cultured in standard culture medium containing 10 mM HEPES, pH 7.0. in glass-bottomed culture dishes (Iwaki) and maintained at 37°C in a humidified chamber. At 24 h after transfection, time-lapse images were acquired in an Axiovert 200 inverted microscope equipped with a charge-coupled device camera (AxioCam; Carl Zeiss, Jena, Germany) using the AxioVision (Carl Zeiss, Jena, Germany) software. Real-time images were acquired every 10 min for 12 h using a standard Rhodamine filter set (excitation at 550 nm and emission at 590–650 nm) at 40× magnification. Short exposure time and a neutral density filter were used during image acquisition to minimize photobleaching and phototoxicity. Digitized images were imported into QuickTime Software (QuickTime v. 5.0; Apple, Cupertino, CA, USA) and converted into movies.

## 3. Results and discussion

### (a) Determination of oligomeric sizes of wild-type and mutant sHSPs

**Figure 1.**
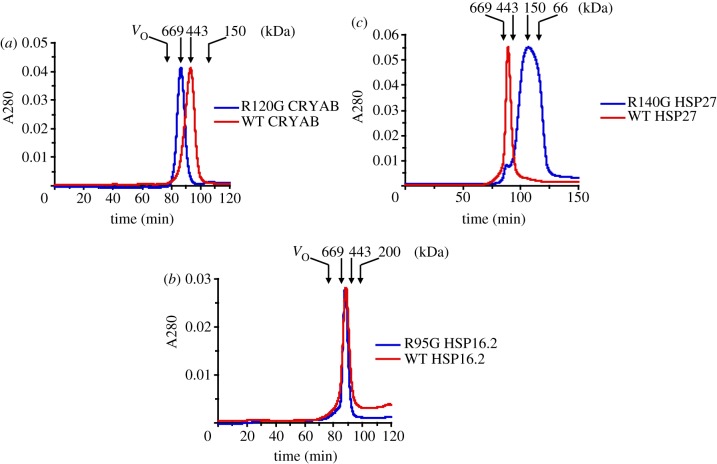
Analysis of oligomeric sizes of WT and mutant sHSPs. SEC was performed using a Superformance column as described in §2d. The elution profiles of WT and mutant (*a*) CRYAB, (*b*) HSP27 and (*c*) HSP16.2 are shown and represent an average of three independent experiments. Elution positions of molecular weight protein standards and their corresponding sizes are indicated on the top of each panel (downward arrows). The position of void volume (*V*_o_) was determined using blue dextran. (Online version in colour.)

Previous studies showed that the R120G mutation in CRYAB altered its secondary, tertiary and quaternary structure [25]. To extend these findings, the corresponding mutations in human HSP27 (R140G) and in *C. elegans* HSP16.2 (R95G) were generated by site-directed mutagenesis and compared with R120G CRYAB by SEC. WT CRYAB (figure 1*a*) and HSP27 (figure 1*b*) eluted at positions corresponding to average molecular sizes of approximately 520 and 560 kDa, respectively (table 1), which is consistent with previous published results [25,61]. The arginine mutation significantly altered the molecular masses of R120G CRYAB and R140G HSP27 (figure 1*a* and table 1), but with opposite consequences. R140G HSP27 formed a polydisperse population of protein oligomers ranging in size from over 600 kDa to approximately 50 kDa (figure 1*b*), which was smaller than the WT HSP27. R120G CRYAB on the other hand was larger (684 kDa) than the WT CRYAB and also appeared no more polydisperse than the WT. There was no major effect upon the assembly of the oligomeric complexes of HSP16.2 when the equivalent arginine residue (R95) was mutated (figure 1*c*). The R95G mutant eluted with almost the same elution volume as that of WT HSP16.2 with an apparent molecular mass of approximately 580 kDa (figure 1*c* and table 1). The three mutants therefore cover the range of potential consequences for the quaternary structure of sHSPs after the introduction of a glycine residue instead of arginine at this conserved site, by either reducing or increasing the oligomer size or producing no apparent change.

**Table 1.** SEC analysis of sHSP oligomers.

molecular masses^a^ of WT sHSPs and their mutants	
WT/mutant sHSP	apparent Mr(kDa)
WT HSP16.2	572 ± 72
R95G HSP16.2	584 ± 80
WT αB-crystallin	520 ± 84
R120G αB-crystallin	684 ± 78
WT HSP27	560 ± 74
R140G HSP27	150 ± 242

^a^Molecular masses (M) were determined from a plot of logM versus *V*_e_/*V*_o_ of molecular mass standards.

### (b) Effect of the sHSP arginine mutations upon their co-sedimentation with intermediate filaments and preventing filament–filament associations

Several *in vitro* assays have been developed to study the effect of the R120G mutation on the interaction of CRYAB with intermediate filaments. These include the ability of sHSPs to co-sediment with intermediate filaments and to prevent filament–filament interactions as detected by falling ball viscometry [51]. Vimentin and GFAP, but not desmin, have been tested in the falling ball assay. Desmin is the physiological target for CRYAB in muscle as revealed by the phenocopying of the disease, desmin-related myopathy, by both desmin and CRYAB mutations [1].

**Figure 2.**
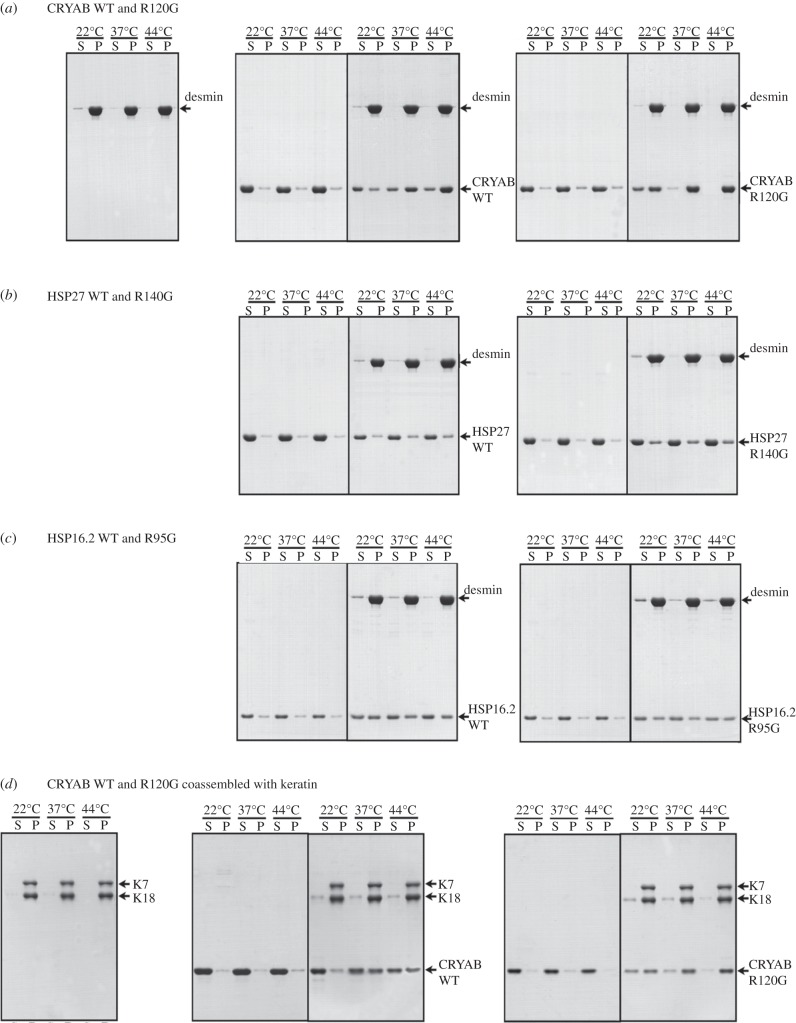
Co-sedimentation of WT and arginine mutant sHSPs to desmin filaments *in vitro*. In this binding assay, the assembly was conducted at temperatures indicated above the relevant gel tracks (*a*–*c*). The pellet (P) and supernatant (S) fractions from the co-sedimentation assays were analysed by SDS-PAGE and visualized by Coomassie Blue staining. The positions of desmin (*a*–*c*), keratin (*d*), WT and arginine mutants of CRYAB (*a*,*d*) HSP27 (*b*) and Hsp16.2 (*c*) are indicated (arrows).

*In vitro* intermediate filament co-sedimentation assays were conducted using optimized pH and salt conditions [26]. Under these conditions, desmin was assembled and sedimented efficiently as shown by the proportion of the protein partitioning into the pellet fraction in the control (figure 2*a*). Both the WT and R120G CRYAB (figure 2*a*) bind to desmin filaments in a temperature-dependent manner. The increased co-sedimentation of R120G CRYAB with desmin filaments compared with WT protein (figure 2*a*) was apparent at all three temperatures. Even at 37°C, almost all of the R120G CRYAB was found to bind to pelletable desmin filaments, whereas the binding of WT protein to desmin was incomplete with a small proportion still remaining in the supernatant fractions (figure 2*a*). For the experiments presented in figure 2*a*, a 1 : 1 molar ratio of desmin : CRYAB was used, although similar results were obtained with decreasing molar ratios at 1 : 0.5, 1 : 0.2 and 1 : 0.1 (data not shown). The binding of HSP27 to desmin filaments (figure 2*b*) was apparently less efficient than HSP16.2 (figure 2*c*) and CRYAB (figure 2*a*). When included at a 1 : 1 molar ratio, the WT HSP27 showed limited binding to desmin filaments with co-sedimentation being greatest at 44°C (figure 2*b*). The co-sedimentation of R140G HSP27 to desmin filaments can be detected at 22°C and this remained unaltered at elevated temperatures (figure 2*b*). The R140G HSP27 appeared to co-sediment more efficiently with desmin filaments than WT HSP27.

In contrast to both CRYAB and HSP27, WT HSP16.2 only partially co-sedimented with desmin filaments (figure 2*c*). We selected this particular sHSP as a representative of those expressed in the animal *C. elegans*. It is a stress-induced sHSP in this animal and multimerization is important to its function [62]. There are also cytoplasmic intermediate filaments in *C. elegans*, albeit quite different in primary sequence to mammalian desmin [63]. The co-sedimentation of HSP16.2 appeared to be independent of temperature, as the WT protein was similarly distributed between supernatant (S) and pellet (P) fractions irrespective of temperature. Similar results were obtained for R95G HSP16.2 (figure 2*c*). Therefore, for this mutant, minimal effect upon the quaternary structure of the protein coincided with little change in the co-sedimentation properties with desmin filaments.

Of the three sHSPs and their respective arginine mutants, it was the R120G CRYAB that was most affected. We therefore considered whether this would alter the co-sedimentation of CRYAB with *in vitro* assembled keratin filaments (figure 2*d*). Filaments of keratins 7 and 18 also co-sedimented with WT CRYAB and co-sedimentation was significantly increased by the R120G CRYAB mutation, mimicking the results obtained with desmin. These data suggest that the increased co-sedimentation of CRYAB R120G is not necessarily restricted to desmin, but includes other type III intermediate filaments, in particular vimentin and GFAP, and also keratins (this study).

To assess the possible function of these various sHSPs and their arginine mutants, an assay to measure the effect of sHSPs upon filament–filament interactions was developed [51]. This assay is based upon falling ball viscometry, which provides a measure of sHSP-desmin interactions at equilibrium. Desmin filaments form a gel capable of supporting the metal ball used in the assay. The effect of the arginine mutations upon the chaperone activity of sHSP was tested. Previous studies showed that the addition of sHSPs, including CRYAB and HSP25, to assembling intermediate filament solutions prevented gel formation and so permitted the ball to sink to the bottom of the tube, even though filament assembly was not inhibited [51]. As expected, after the assembly of desmin, a gel formed preventing the ball from falling to the bottom of the tube. The presence of WT human CRYAB, *C. elegans* HSP16.2 and HSP27 with desmin allowed the ball bearing to sink to the bottom of the capillary (table 2). These WT sHSPs are apparently very effective at preventing gel formation over a 10-fold concentration range (table 2). The R95G mutation did not abolish the activity of HSP16.2 in this assay. In contrast, the mutant R120G CRYAB appeared completely ineffective at inhibiting gel formation, the ball remaining on top of the assembled desmin sample in the capillary. HSP27 R140G was equally effective as the WT protein at 1 : 1 ratios, but was ineffective at the 0.2 : 1 ratio, in contrast to the WT HSP27 (table 2).

**Table 2.** Summary of the data collected for the effect of sHSPs and their arginine mutants on the gel formation by desmin filaments as monitored by the falling ball assay. Desmin can form a protein gel capable of supporting a small stainless steel ball. Addition of WT sHSPs in a range of molar ratios from 1 : 1 to 0.1 : 1 to desmin allow filament assembly but then prevent gel formation. In this case, the ball will descend easily into the bottom of the capillary tube. A similar result was obtained for R95G HSP16.2. The R120G CRYAB abrogated this activity of αB-crystallin even over a 10-fold concentration range. The inhibitory effect of R140G HSP27 on gel formation is compromised, but not completely abolished, as this mutant can still prevent gel formation when added at a 1 : 1 molar ratio.

WT and mutant sHSPs added to assay	desmin gel formation as indicated by the ball position		
sHSP : desmin (molar ratio)	1 : 1	0.2 : 1	0.1 : 1
WT CRYAB	bottom	bottom	bottom
R120G CRYAB	top	top	top
WT HSP27	bottom	bottom	bottom
R140G HSP27	bottom	top	top
WT HSP16.2	bottom	bottom	bottom
R95G HSP16.2	bottom	bottom	bottom

This assay provides a rapid way to assess the potential activity of different intermediate filament and sHSP combinations. In table 3, we provide additional evidence that sHSPs from evolutionary-unrelated organisms appear to have an innate ability to affect desmin gel formation in this falling bead assay. As an extreme test of this concept, the chloroplast-specific sHSP, HSP21 [64], was found to be able to prevent desmin gel formation. It was therefore surprising that another potential desmin-interacting mammalian sHSP, HSP20, is not equivalent to CRYAB in this assay. HSP20 is involved in cardioprotection and can also coassemble with both HSP27 and CRYAB [65,66]. These data suggest that while sHSPs from distant organisms can affect gel formation, the different mammalian sHSPs do not possess completely equivalent properties.

**Table 3.** Effect of WT and mutant sHSPs on the gel formation by desmin filaments as monitored by the falling ball assay. Inter-filament interactions between assembled desmin filaments lead to the formation of a protein gel that is capable of supporting a small stainless steel ball. Addition of sHSPs in a molar ratio of 1 : 1 to desmin prevented this gel formation, thus allowing the ball to drop to the bottom of the capillary tube. Carbonic anhydrase was used as a control in these experiments.

sHSPs added to desmin solution	ball position
no addition	top
carbonic anhydrase	top
CRYAB (HSPB5)	bottom
HSP21 (*Arabidopsis thaliana*)	bottom
HSP20 (HSPB6)	top

### (c) Measuring CRYAB interactions with type III intermediate filament proteins using non-equilibrium and equilibrium methods

**Figure 3.**
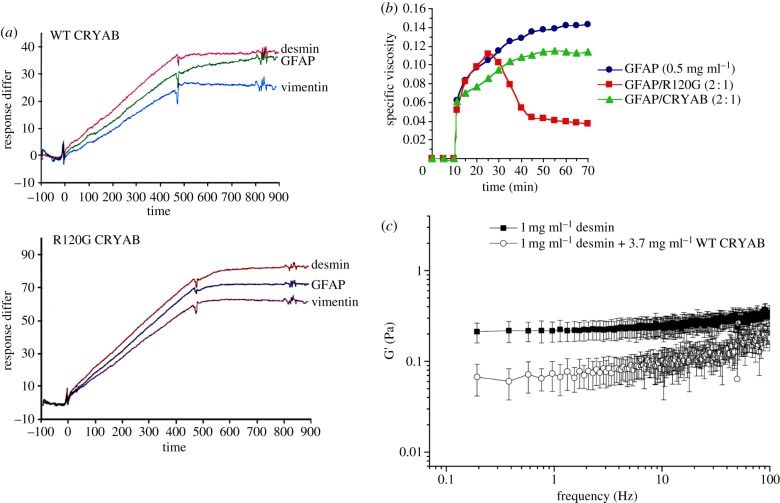
Equilibrium methods of detecting the interaction of CRYAB with intermediate filaments. (*a*) Binding kinetics of WT and R120G CRYAB to different intermediate filament (IF) proteins as measured by SPR. Desmin, GFAP and vimentin were immobilized to the dextran matrix of a CM5 sensor chip. Binding of 20 g ml WT (*a*) or R120G (*b*) CRYAB to immobilized IF proteins were monitored at 37°C for 7.5 min, followed by washing for 7.5 min. Measurements were performed at a flow rate of 5 l min^–1^ using a Biacore 3000 Biosensor. Resonance measured during 900 s of exposure is plotted on the ordinate in arbitrary units. (*b*) Viscometric analyses of GFAP assembly in the absence or presence of CRYAB. GFAP (0.5 mg ml^−1^) was assembled either alone or coassembled with either WT or R120G CRYAB in a molar ratio of 1 : 2 at 37°C. Assembly was initiated at the 10 min time-point by addition of a 20-fold concentrated assembly buffer. Specific viscosity was measured 1 min after assembly and then at 5 min intervals for 60 min. The profile obtained for GFAP reflects the normal increase of specific viscosity after initiation of filament assembly. In the presence of WT CRYAB, GFAP exhibited a slightly decreased specific viscosity over a period of 40–45 min before reaching a plateau. In contrast, for GFAP assembled in the presence of R120G CRYAB, the specific viscosity increased normally only during the first 25 min. Afterward it decreased dramatically, indicative of GFAP filament aggregation. (*c*) Passive rheology measurements by one-particle microrheology. WT desmin was assembled alone or with a 1 : 10 molar ratio of WT desmin: WT CRYAB. The position fluctuations of beads immersed in the network were measured by trapping beads with a weak 808 nm laser and measuring scattered light on a QPD. The elastic modulus was corrected for the presence of the optical trap. Average G’ values (Pa) ±1 s.d. from five independently trapped beads for WT desmin and from 10 independently trapped beads for WT desmin with WT CRYAB are plotted against frequency (Hz). Desmin assembled alone has a higher stiffness compared with when WT CRYAB is present. This difference is statistically significant with *p* ≤ 0.01, using a type 2, one-tailed Student's *t*-test. (Online version in colour.)

SPR provides an equilibrium method to assess the relative binding of WT and R120G CRYAB to desmin, GFAP and vimentin (figure 3*a*). It can be seen that both WT and R120G CRYAB had a greater capacity for binding desmin than the other type III intermediate filament proteins, vimentin or GFAP. Moreover, there was a significant increase in the binding of R120G CRYAB to all three type III intermediate filament proteins. These data support the interpretation that there is selectivity in CRYAB binding to type III intermediate filament proteins and that the R120G mutation increases the binding of CRYAB to intermediate filaments supporting the *in vitro* co-sedimentation data and electron microscopy data [25,26]. More detailed interpretation of these data is complicated by the uncertainty over which intermediate filament assembly form is bound to the chip surface given that, at 20 µg ml^−1^, both desmin [67] and vimentin [68,69] have the potential to form filaments while this is likely not the case for GFAP, which has a critical concentration of 80 µg ml^−1^ [70]. Purging the chip with 6 M guanidine hydrochloride should ensure that only intermediate filament monomers are bound to the chip surface. On-chip association of subunits, however, is a possibility once binding-buffer conditions are restored. Furthermore, the inherent polydispersity of CRYAB [71] further complicates the interpretation as it is not known whether there is any selectivity by specific CRYAB oligomers for intermediate filaments. SPR has been used to study CRYAB subunit dynamics [72] and to assess the relative affinity of different client proteins [73]. These data confirm the preference of CRYAB for desmin and, from the dissociation kinetics, it is clear that once bound, CRYAB dissociates slowly from all three type III intermediate filament proteins. This would fit the conclusion that there is a high affinity binding site for CRYAB clients [74] and that topologically distinct sites are present on CRYAB [75] for these different clients as born out by peptide array studies [76]. With respect to a protein polymer such as an intermediate filament, the rules for client selection by sHSPs may not parallel those established for monomeric, destabilized clients such as T4 lysozyme [75,77]. Indeed there is no evidence currently to suggest that intermediate filament proteins naturally adopt unfolded conformations within the filament, although this can be induced by mechanical stretching [78]. There is, however, evidence to suggest that filaments exhibit different subunit geometries that can be differentially detected by antibodies [79,80] and could be induced by post-translational modifications [19,81–87]. The presence of disease-causing mutants or GFP-tagged intermediate proteins is sufficient to increase the binding of CRYAB to the filaments [58,88], which we interpret as evidence of altered subunit geometries inducing sHSP association with intermediate filaments.

Using Ostwald viscometry, the assembly of GFAP in the presence of WT and R120G CRYAB was analysed (figure 3*b*). Addition of CRYAB reduced the overall viscosity of the solution, but the presence of the R120G CRYAB induced a catrastrophic loss of viscosity after approximately 25 min when aggregates were visible in the solution consistent with the falling ball assay. We confirmed the presence of highly bundled filaments by electron microscopy (see the electronic supplementary material, figure S1). These data indicate the ability of WT CRYAB to reduce the apparent viscosity of a solution of GFAP filaments. In contrast, the presence of R120G CRYAB promotes interactions between GFAP filaments, which induces clumping and their eventual aggregation, which has the effect of significantly reducing solution viscosity, but by a completely different mechanism and with greater impact than the viscosity reduction induced by WT CRYAB. The effect of the R120G mutation is to drive the aggregation of the GFAP filaments.

A disadvantage of the Ostwald viscometer is that flow-induced filament orientation occurs during the measurements, which may lead to shear-thinning. For this reason, we turned to a passive rheology approach using optically trapped beads (figure 3*c*). As intermediate filaments are semi-flexible polymers that show both elastic and viscous properties, bulk rheology has been used to assess the mechanical properties of desmin networks in terms of their flexibility and persistence lengths [89]. We find that CRYAB slightly reduces the elastic modulus of the desmin filament networks as measured by one-particle passive microrheology (figure 3*c*). This confirms the interaction of CRYAB with intermediate filaments, eliciting a measurable change in the biomechanical properties of the filament solution. The divalent cation-mediated interaction of C-terminal sequences of the type III intermediate filament proteins could partly explain the filament solution properties [90] and parallels the similar role for the C-terminal extensions in neurofilaments [91]. The precise details of how CRYAB might prevent the filament–filament interactions as measured by the falling ball (table 2; [51]), low-speed sedimentation [26,52] and viscosity assays (figure 3*b*) is not yet determined and neither is the question of how this relates to the observed reduction in the stiffness of the desmin filament network. Nevertheless, for other poly-electrolyte systems such as actin [92] and neurofilaments [91,93], the cations and pH in solution and the amino acid sequence exposed at the filament surface are known to be key factors in driving gel formation, sHSP association and thus sample stiffness.

### (d) Temperature and pH dependency of the interaction of CRYAB with desmin, GFAP and vimentin intermediate filaments

**Figure 4.**
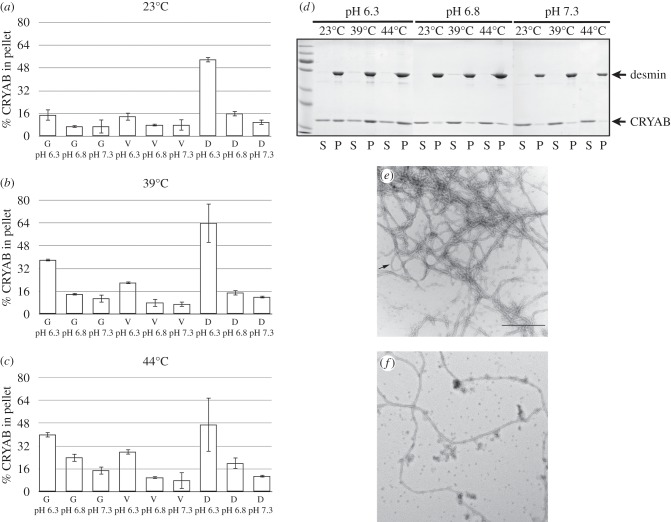
CRYAB interaction with desmin is divalent cation-, pH- and temperature-dependent. Binding of CRYAB to desmin, GFAP and vimentin filaments was assessed by high-speed co-sedimentation assay (*a*–*d*). The quantified results are presented as the mean±s.d. of the percentage of CRYAB that had co-sedimented with WT desmin, GFAP and vimentin at 23°C, 39°C and 44°C (*a*–*c*, respectively). Note, most binding occurs at pH 6.3 and at 39°C and 44°C with a preference for desmin over vimentin and GFAP. A representative gel image used for the quantification of binding to desmin is shown (*d*). Three independent experiments were used for the quantifications. (*e*,*f*) WT desmin formed filaments of a few micrometres in length and had a much higher density of CRYAB particles (arrow) surrounding the filaments at pH 6.3 (*e*) compared with pH 7.3 (*f*). Images are taken at the same magnification. Scale bar, 500 nm. G, GFAP; V, vimentin; D, desmin; S, supernatant; P, pellet.

Desmin, GFAP and vimentin were assembled *in vitro* at three different pH values and temperatures to see how these variables influenced CRYAB association. Previous studies with recombinant purified human intermediate filament proteins have looked at temperature effects (e.g. [67]), but not pH effects, although the CRYAB association with a mixture of desmin and actin at different pHs was investigated previously [94]. Intermediate filaments were formed at all pHs and temperatures analysed (figure 4*e*,*f*; data not shown for GFAP and vimentin). Desmin filaments were coated with CRYAB particles at pH 6.3 and at 23°C (figure 4*e,f*). High-speed co-sedimentation analyses showed that at pH 6.3 and over the temperature range from 39°C to 44°C, more than 35 per cent of the total CRYAB was associated with the desmin and GFAP filaments. In line with the SPR data, CRYAB binding followed the same trend, with desmin binding the most and vimentin the least (figure 4*a–c*) at the temperatures and pHs investigated. Lower binding was observed at pH 7.3 and 6.8 (figure 4*a*–*c*) indicating that there was a pH dependency in CRYAB binding to desmin, GFAP and vimentin. We also investigated the pH effects on the binding of CRYAB to preformed GFAP filaments by co-sedimentation and observed a similar trend of increased binding at pH 6.3 that was also temperature-dependent (data not shown). Similar studies were not possible for desmin, as in the absence of CRYAB desmin filaments were attracted to the plastic surfaces of tips and tubes (data not shown).

Ischaemia results in a pH decline in muscle tissue [95] and the translocation to the Z- and I-bands of resident sHSPs [96,97], which is the location of the desmin intermediate filaments. Therefore, the data we have presented evidence the importance of pH changes to the interaction of CRYAB with desmin. The structure of CRYAB would be expected to change in acidosis, resulting in a dimer-monomer transition of the α-crystallin domain [98,99] as well as its activation [100]. The R120G mutation in CRYAB, in contrast, stabilized its dimers at low pH [101], the result of removal of a positive charge from within the dimer interface which contains histidines sensitive to physiological pH changes [44,98]. Therefore, it appears that acidic pH can induce the release of monomeric WT CRYAB, but not the R120G mutant. This perhaps leads to extended interactions with desmin filaments, compared with WT CRYAB, at physiological pH values as evidenced by the studies here and those previously published [25,26,102].

### (e) Transient transfection studies

In order to determine the effect of these arginine mutations upon the potential *in vivo* activity of the respective sHSPs, transient transfection assays were performed. Desmin filaments form characteristic aggregates as part of the histopathology in the cardiomyopathies so far described with the R120G CRYAB mutation. As a mimic of this situation, desmin was co-transfected with either the WT or mutant sHSP and the formation of desmin aggregates compared with that of desmin alone. The human cell line H36CE2 was chosen because it is a human cell line that does not express desmin, but does express vimentin. Lens epithelial cells can express desmin as part of their response to posterior capsule opacification [103]. The results were recorded as the percentage of cells with desmin-positive aggregates.

**Figure 5.**
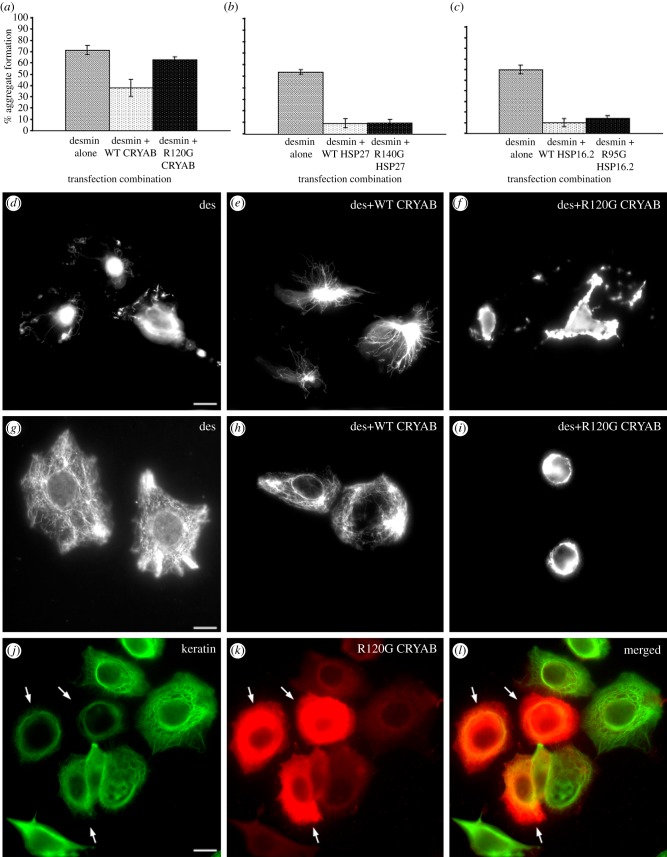
Expression of R120G CRYAB induces the aggregation of intermediate filaments. (*a*–*c*) The immortalized human lens epithelial cell line H36CE2 was transiently transfected with combinations of desmin with either WT CRYAB or R120G CRYAB (*a*), with desmin and either WT HSP27 or R140G HSP27 (*b*) and with desmin and either WT HSP16.2 or R95G HSP16.2 (*c*). The number of desmin aggregate-containing cells were then counted and the mean ± s.d. calculated and plotted as bar charts. R120G CRYAB was the only mutant sHSP that significantly increased the number of desmin aggregates in the transiently transfected cells. Representatives of transfected cells are not shown. (*d*–*f*). (*d*) SW13 Vim^−^ cells were transiently co-transfected with desmin alone, (*e*) desmin and WT CRYAB and (*f*) desmin and R120G CRYAB. Note the aggregates in those cells co-transfected with desmin and R120G CRYAB. (*g*–*i*) The effect of the transient transfection of WT CRYAB and R120G CRYAB upon the endogenous keratin networks in MCF7 cells. (*g*) MCF7 cells were transiently co-transfected with desmin alone, (*h*) desmin and WT CRYAB and (*i*) desmin and R120G CRYAB. (*j*–*k*) Transient transfection of MCF7 cells with R120G CRYAB. (*j*) Cells were then probed with both keratin and (*k*) CRYAB antibodies. Note that in the R120G CRYAB transfected cells the endogenous keratin networks have collapsed around the nucleus, indicating the ability of CRYAB R120G to act in a dominant negative fashion to aggregate keratin filaments. Scale bar, 10 µm.

The transfection of desmin into H36CE2 cells leads to the formation of desmin-positive aggregates in the cytoplasm of a high proportion of the cells. Co-transfection with CRYAB significantly reduced the incidence of aggregate-positive cells, but when the R120G CRYAB was co-transfected with desmin, this positive effect was lost and desmin aggregates were once again apparent (figure 5*a*). In contrast, the co-transfection of either HSP27 or R140G HSP27 (figure 5*b*) or HSP16.2 or R95G HSP16.2 (figure 5*c*) all significantly reduced desmin aggregate formation in transfected H36CE2 cells.

We selected two other cell lines to confirm the tendency of R120G CRYAB to induce the aggregation of desmin filaments in transiently transfected cells. In SW13/Cl2 Vim^−^ cells, an adenocarcinoma cell line that lacks cytoplasmic intermediate filaments, the transiently transfected desmin also failed to form effective networks of desmin filament (figure 5*d*). Only when co-transfected with WT CRYAB (figure 5*e*) were desmin filament networks observed. As with H36CE2 cells, co-transfection of desmin with R120G CRYAB induced desmin filament aggregation (figure 5*f*). These aggregates costained with CRYAB antibodies (data not shown). A similar experimental series is also shown for MCF7 cells, which have an endogenous cytoplasmic keratin network, showing similar results for co-transfection of WT and R120 CRYAB, albeit in these cells desmin alone was capable of forming a filament network (figure 5*g*–*i*). The co-transfection of R120G CRYAB with desmin caused the perinuclear collapse of the desmin filaments (figure 5*i*). Interestingly, the transient transfection of R120G CRYAB into MCF7 cells was also capable of causing the collapse of the endogenous keratin network of filaments (figure 5*j*,*k*), but there was no loss in cell viability.

**Figure 6.**
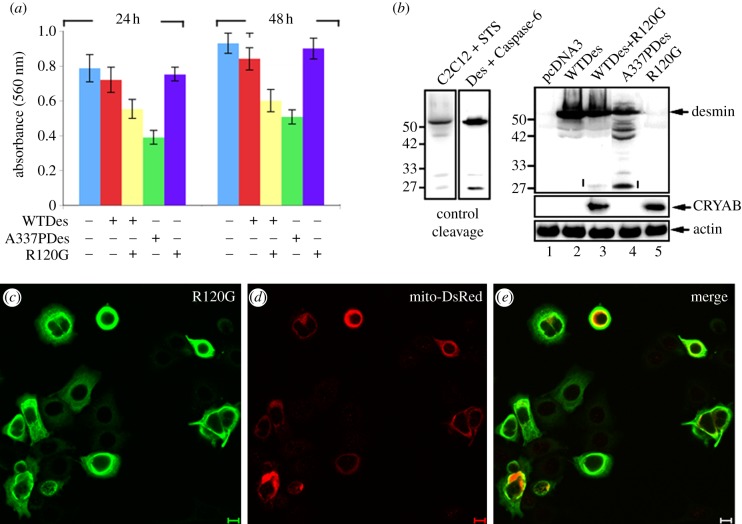
Expression of mutant desmins reduces viability of MCF7 cells. (*a*) Cell viability was measured in MCF7 cells transiently transfected with WT desmin, A337P desmin, R120G CRYAB alone or co-transfected with WT desmin and R120G CRYAB at a 1 : 1 molar ratio. Cells transfected with empty pcDNA3.1 vector were used as a control. The viability of transfected MCF7 cells was determined at 24 and 48 h using the MTS-based cell viability assay. Data shown are the mean±s.e. from three independent experiments. Statistical significance was analysed by one-way ANOVA and the level of significance was set at *p* ≤ 0.05. (*b*) Desmin fragmentation by caspases. Two desmin fragments (arrowheads) were generated in C2C12 cells induced to undergo apoptosis by treatment with 1 μM staurosporine (C2C12+STS) and *in vitro* by cleavage of purified desmin with active caspase 6 (Des + Caspase 6). One of the proteolytic fragments was faintly observed in cells co-transfected with WT desmin and R120G αB-crystallin (lane 3). Levels of protein expressed in transfected MCF7 cells were determined at 48 h after transfection. Immunoblotting of total cell lysates and probing with antibodies to desmin, CRYAB and finally actin, as a loading control. (*c*–*e*) To determine the effect of expressing R120G CRYAB (green channel) upon the distribution of mitochondria, MCF7 cells were transiently transfected with an IRES-containing bicistronic vector that allows simultaneously expression of both mitochondrially targeted red fluorescent protein (*c*; red channel) and R120G CRYAB (*d*; green channel). Note the collapse of the mitochondria into perinuclear locations in those cells (*e*; merged red and green channels) expressing R120G CRYAB.

In the course of these experiments involving MCF7 cells, we noticed low transfection rates for the desmin when co-transfected with R120G CRYAB. We therefore monitored cell viability after transfection. We included a series of controls including the DRM-causing desmin mutant A337P. The results are presented in figure 6*a*. Co-transfection of R120G CRYAB with WT desmin induced a significant reduction in cell viability. This was not observed when R120G CRYAB was transiently transfected into MCF7 cells. Neither was the transfection of WT desmin deleterious for cell viability. Interestingly, only the DRM-causing desmin mutant A337P induced a similar reduction in cell viability. These data suggest that DRM-causing mutations in either desmin (A337P) or CRYAB (R120G) were capable of reducing cell viability, but R120G CRYAB was dependent upon the presence of desmin for this effect.

Desmin has been found to propagate the canonical apoptosis pathway, it being a substrate for caspase 6 [9] and evidenced by our control experiments using staurosporin-treated C2C12 cells and caspase 6-treated desmin (figure 6*b*). Similar-sized desmin proteolytic fragments were observed in samples prepared from MCF7 cells transfected with A337P desmin and for those co-transfected with the combination of WT desmin and R120G CRYAB (figure 6*b*). Using the modified IRES-Mito-DsRED-based pcDNA3.1 vector to identify the mitochondria of transient transfected cells, R120G CRYAB induced the collapse of mitochondria along with the perinuclear aggregation of the endogenous keratin filaments (figure 6*c*–*e*), but as evidenced by the cell viability assay (see the electronic supplementary material, figure S2) the expression of disease-causing desmin mutants induces apoptosis by the activation of caspases. The fact that R120G CRYAB induces the collapse of the endogenous keratin filaments and the mitochondria in MCF7 cells singly transfected with just R120G CRYAB is very important especially as these are phenocopied by the co-transfection of both R120G CRYAB with desmin. The important difference is that only when R120G CRYAB is co-transfected with WT desmin is there a significant decrease in cell viability, which we take as prime fascia evidence of a unique and specific interaction that sets it apart from keratins and vimentin, the intermediate filaments found in H36CE2 and MCF7 cells.

### (f) CRYAB and desmin form a functional complex

The transient transfection studies suggest that desmin and CRYAB are indeed a functional complex, which can determine whether transiently transfected cells continue to proliferate or die (figure 6). Analysis of transgenic animal models have tended to overlook the functional aspect of the desmin-CRYAB interaction, focusing rather on the amyloid-forming potential of R120G CRYAB [5], or on the ability of R120G CRYAB [104,105] and desmin mutants [7] to inhibit the proteosome or affect autophagy [8,38,106]. Our studies illustrate that, while these are important consequences, the primary lesion in the development of DRM appears to be the dominant negative effect of the desmin–CRYAB complex on cell viability. Indeed the effects of CRYAB on MCF7 cells suggests that the only muscle-specific factors required in order to precipitate the loss of viability is either a mutant desmin, or R120G CRYAB in the presence of WT desmin. This therefore suggests a very simple explanation for why R120G CRYAB induces a myopathy and not, for instance, a neuropathy, as muscle is the tissue where there is significant expression of desmin, the intermediate filament partner needed to trigger cell death and the muscle pathology [9,18]. Indeed our data also suggest that aggregation of the resident keratin intermediate filament network by R120G CRYAB and the concentration of the mitochondria in a perinuclear region are insufficient triggers for this loss in MCF7 viability. The fact that mutations in either desmin [2] or CRYAB [1] phenocopy each other further evidences this key functional link, which we suggest is fundamental to the muscle pathologies associated with the respective mutations in CRYAB and desmin. We conclude that the specific intermediate filament expression pattern must be considered as a key factor in any sHSP-based pathology and this study is further evidence of the functional importance of the sHSP-intermediate filament protein complex [41,42].
